# The Metabolic Fate of *ortho*-Quinones Derived from Catecholamine Metabolites

**DOI:** 10.3390/ijms17020164

**Published:** 2016-01-27

**Authors:** Shosuke Ito, Yuta Yamanaka, Makoto Ojika, Kazumasa Wakamatsu

**Affiliations:** 1 Department of Chemistry, Fujita Health University School of Health Sciences, Toyoake, Aichi 470-1192, Japan; sito@fujita-hu.ac.jp (S.I.); fuh-tech.mau-in.yu.0831@qc.commufa.jp (Y.Y.); 2 Department of Applied Molecular Biosciences, Graduate School of Bioagricultural Sciences, Nagoya University, Nagoya, Aichi 464-8601, Japan; ojika@agr.nagoya-u.ac.jp

**Keywords:** catechols, *ortho*-quinones, quinone methide, catecholamines, neuromelanin

## Abstract

*ortho*-Quinones are produced *in vivo* through the oxidation of catecholic substrates by enzymes such as tyrosinase or by transition metal ions. Neuromelanin, a dark pigment present in the *substantia nigra* and *locus coeruleus* of the brain, is produced from dopamine (DA) and norepinephrine (NE) via an interaction with cysteine, but it also incorporates their alcoholic and acidic metabolites. In this study we examined the metabolic fate of *ortho*-quinones derived from the catecholamine metabolites, 3,4-dihydroxyphenylethanol (DOPE), 3,4-dihydroxyphenylethylene glycol (DOPEG), 3,4-dihydroxyphenylacetic acid (DOPAC) and 3,4-dihydroxyphenylmandelic acid (DOMA). The oxidation of catecholic substrates by mushroom tyrosinase was followed by UV-visible spectrophotometry. HPLC analysis after reduction with NaBH_4_ or ascorbic acid enabled measurement of the half-lives of *ortho*-quinones and the identification of their reaction products. Spectrophotometric examination showed that the *ortho*-quinones initially formed underwent extensive degradation at pH 6.8. HPLC analysis showed that DOPE-quinone and DOPEG-quinone degraded with half-lives of 15 and 30 min at pH 6.8, respectively, and >100 min at pH 5.3. The major product from DOPE-quinone was DOPEG which was produced through the addition of a water molecule to the quinone methide intermediate. DOPEG-quinone yielded a ketone, 2-oxo-DOPE, through the quinone methide intermediate. DOPAC-quinone and DOMA-quinone degraded immediately with decarboxylation of the *ortho*-quinone intermediates to form 3,4-dihydroxybenzylalcohol (DHBAlc) and 3,4-dihydroxybenzaldehyde (DHBAld), respectively. DHBAlc-quinone was converted to DHBAld with a half-life of 9 min, while DHBAld-quinone degraded rapidly with a half-life of 3 min. This study confirmed the fact that *ortho*-quinones from DOPE, DOPEG, DOPAC and DOMA are converted to quinone methide tautomers as common intermediates, through proton rearrangement or decarboxylation. The unstable quinone methides afford stable alcoholic or carbonyl products.

## 1. Introduction

Neuromelanin is a complex polymeric compound present in the human central nervous system which is synthesized mainly in dopaminergic neurons of the *substantia nigra* and in noradrenergic neurons of the *locus coeruleus* [1,2]. The synthesis and accumulation of neuromelanin inside neurons arises during brain aging and there is evidence that neuromelanin is involved in the pathogenesis of neurodegenerative diseases such as Parkinson’s disease [3,4,5]. Notably, the *substantia nigra* and *locus coeruleus* are the areas in the brain with the highest concentration of neuromelanin pigment [6].

**Figure 1:**
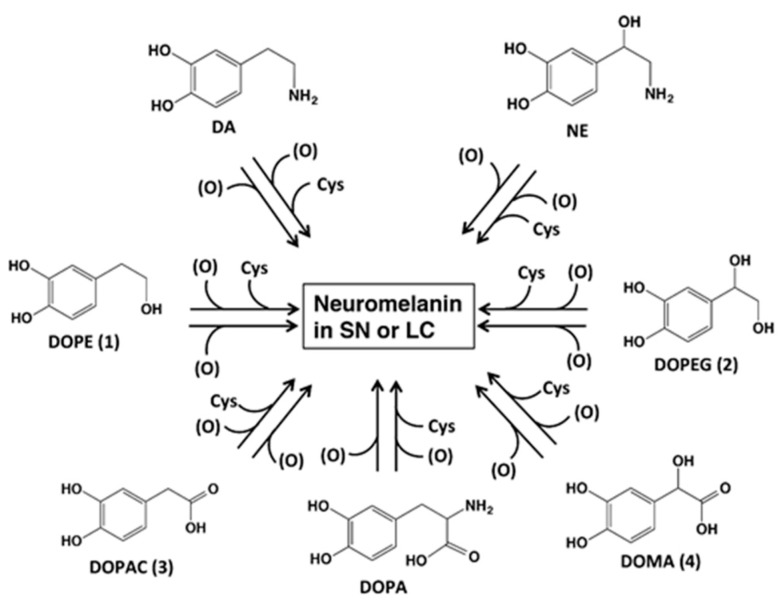
Possible participation of catecholic metabolites known to be present in various regions of the brain that may be incorporated into neuromelanin [7,8]. In addition to dopamine (DA) and norepinephrine (NE) and the corresponding Cys-derivatives, these other metabolites are also thought to be incorporated into neuromelanin [9,10]. *substantia nigra*, SN; *locus coeruleus*, LC; 3,4-dihydroxyphenylethanol, DOPE; 3,4-dihydroxyphenylethylene glycol, DOPEG; 3,4-dihydroxyphenylacetic acid, DOPAC; 3,4-dihydroxyphenylmandelic acid, DOMA; 3,4-dihydroxyphenylalanine, DOPA; cysteine, cys. The (O) represents the oxidants. Adapted from [10] with minor modifications.

The biosynthesis and structure of neuromelanin remains poorly understood, mainly because of difficulties in its isolation and the complexity of its structure [1,2]. Nevertheless, our degradative approach suggested that the pigmented part of *substantia nigra* neuromelanin is derived from dopamine (DA) and cysteine in a molar ratio of 2:1 [7,8]. In addition, it was recently suggested that various catecholic metabolites are incorporated into neuromelanin from the *substantia nigra* and *locus coeruleus*, including 3,4-dihydroxyphenylalanine (DOPA), 3,4-dihydroxyphenylethanol (DOPE, **1**), 3,4-dihydroxyphenylethylene glycol (DOPEG, **2**), 3,4-dihydroxyphenylacetic acid (DOPAC, **3**) and 3,4-dihydroxymandelic acid (DOMA, **4**), which are metabolites of dopamine (DA) and norepinephrine (NE) formed by the oxidative deamination by monoamine oxidase followed by reduction/oxidation (Figure 1) [9,10,11].

Oxidation of those catecholamine metabolites leads to the production of *ortho*-quinones, although the nature of the oxidants remains controversial [12]. Once formed, the *ortho*-quinones are trapped rapidly by cysteine (or other thiols) as long as it is present [13]. The cysteine adducts are oxidized to give rise to pheomelanic pigments [9,10]. After consumption of available cysteine in neurons, *ortho*-quinones derived from DA, NE and DOPA are cyclized to give aminochromes and eventually eumelanic pigments [9,10,13]. Thus, the production of neuromelanin is considered as a detoxifying mechanism of otherwise toxic *ortho*-quinones [14]. The fate of *ortho*-quinones derived from the catecholamine metabolites DOPE (**1**), DOPEG (**2**), DOPAC (**3**) and DOMA (**4**) are studied by kinetic and spectroscopic techniques and the results are presented in this paper.

In this study, we compared the reactivities of those *ortho*-quinones regarding their half-lives and degradation products. *ortho*-Quinones are produced by oxidation with mushroom tyrosinase. The fates of *ortho*-quinones were examined by two methods [15,16]: UV-visible spectrophotometry to detect *ortho*-quinones with an absorption maximum around 400 nm and HPLC to follow the degradation of *ortho*-quinones and the appearance of products after reduction to the corresponding catechols by NaBH_4_ or ascorbic acid. The results suggest that *ortho*-quinone products from catecholamine metabolites may not be incorporated into neuromelanin, but exert neurotoxicity as such because *ortho*-quinones are highly reactive compounds that lead to cytotoxicity through their binding to thiol enzymes and the production of reactive oxygen species [17,18,19].

## 2. Results

### 2.1. Spectrophotometric Examinations

The UV-visible spectrophotometric examinations were performed at a 100 µM concentration of the four catecholamine metabolites in 50 mM sodium phosphate buffer, pH 6.8 or 5.3, at 37 °C. The oxidation was initiated by adding 50 U/mL mushroom tyrosinase and was then periodically followed.

**Figure 2:**
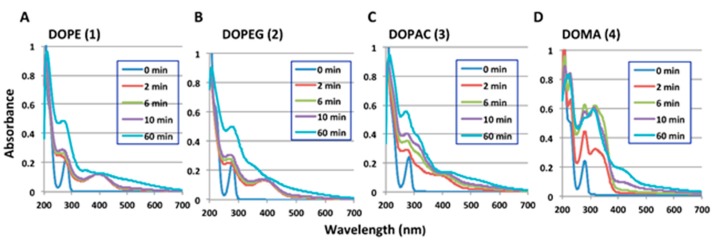
Time course of the degradation of *ortho*-quinone products from catecholamine metabolites, (**A**) DOPE (**1**); (**B**) DOPEG (**2**); (**C**) DOPAC (**3**); (**D**) DOMA (**4**). UV-visible spectral changes associated with the tyrosinase catalyzed oxidation of different catechols at pH 6.8. Each experiment was performed twice with similar results.

Spectral changes at pH 6.8 are shown in Figure 2. DOPE (**1**) and DOPEG (**2**) were oxidized to the corresponding *ortho*-quinones (400 nm) in 2 min and were then gradually degraded to ill-defined products after 60 min (Figure 2A,B). DOPAC (**3**) was converted to an *ortho*-quinone (400 nm) in 2 min but was rapidly degraded with a broader absorption (Figure 2C). DOMA (**4**) was oxidized to an *ortho*-quinone having an absorption maximum at 420 nm which may correspond to the *ortho*-quinone oxidation product of 3,4-dihydroxybenzaldehyde (DHBAld (**7**); Figure 2C,D; see below for the identification). Spectral changes at pH 5.3 are shown in Figure S1. At pH 5.3, the *ortho*-quinones formed were much more stable than at pH 6.8 and showed more subtle spectral changes in 60 min.

### 2.2. HPLC Examinations

**Figure 3:**
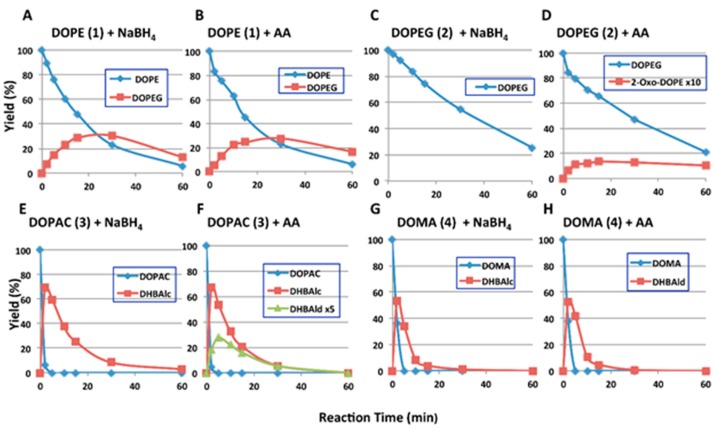
HPLC following the degradation of *ortho*-quinone products from catecholamine metabolites at pH 6.8. (**A**,**B**) DOPE (**1**); (**C**,**D**) DOPEG (**2**); (**E**,**F**) DOPAC (**3**); (**G**,**H**) DOMA (**4**). For **A**, **C**, **E** and **G**, the oxidation was stopped by the addition of NaBH_4_; For **B**, **D**, **F** and **H**, the oxidation was stopped by the addition of ascorbic acid (AA). For the sake of clarity, the yields of DOPEG ketone (**5**) were multiplied by a factor of 10 in (**D**) and those of DHBAld (**7**) were by a factor of 5 in (**F**).

HPLC examinations were performed for the products after reduction of *ortho*-quinones with NaBH_4_ or ascorbic acid to the corresponding catechols (Figure 3 and Figure S2). The difference between reduction with NaBH_4_ and with ascorbic acid is that NaBH_4_ reduces not only the *ortho*-quinone moiety but also the carbonyl group to a hydroxyl group, whereas ascorbic acid is able to reduce only the former. HPLC conditions for the product analysis are summarized in Table 1.

**Table 1:** Half-lives of *ortho*-quinone products from catecholamine metabolites (**1**–**4**) and HPLC conditions used to follow the oxidation.

*Ortho*-Quinone Derived from	Half-Life (*t*_1/2_, min)				HPLC Conditions ^1^
	with NaBH_4_		with Ascorbic Acid		
	pH 6.8	pH 5.3	pH 6.8	pH 5.3	
DOPE (**1**)	14.6	128	15.2	114	80:20, 35 °C
DOPEG (**2**)	30.9	187	30.0	154	90:10, 35 °C
DHBAlc (**6**) from DOPAC (**3**)	9.1	46.8	9.5	47.8	85:15, 50 °C
DHBAld (**7**) from DOMA (**4**)	3.0 ^2^	3.3 ^2^	3.0 ^2^	3.3 ^2^	85:15, 50 °C

^1^ The mobile phase of 0.4 M formic acid:methanol, *v*/*v* (%), and the column temperature; ^2^ These values were subject to experimental errors because of the rapid degradation of DHBAld (**7**)—quinone. DHBAlc, 3,4-dihydroxybenzylalcohol; DHBAld, 3,4-dihydroxybenzaldehyde.

HPLC following the oxidation of DOPE (**1**) at pH 6.8 showed a gradual degradation of DOPE (**1**)-quinone (analyzed as DOPE (**1**) after reduction with ascorbic acid (AA) or NaBH_4_) with production of a more hydrophilic compound with a shorter retention time compared to DOPE (**1**) (Figure 3A,B). The compound was found to have a UV absorption spectrum almost identical to DOPE (**1**) but different from the 3,4,6-trihydroxy chromophore of 6-hydroxydopamine (Figure S3A). It was then identified as DOPEG (**2**) by co-injection on HPLC.

When analyzed after ascorbic acid reduction, the oxidation of DOPEG (**2**) gave a product with a longer retention time than DOPEG (Figure 3D). The compound was found to have a UV absorption spectrum almost identical to DHBAld (**7**) (Figure S3B), suggesting it to have a carbonyl group adjacent to the 3,4-dihydroxyphenyl ring. To confirm its structure, the compound was prepared by NaIO_4_ oxidation of DOPEG (**2**) and isolated, although in a low yield (1%), by preparative HPLC. The compound was reduced by NaBH_4_ to afford DOPEG (**2**) (Figure S4), suggesting that it is 2-oxo-DOPE (**5**). This identification was then confirmed by direct comparison with an authentic sample (see below). When analyzed after NaBH_4_ reduction, the 2-oxo-DOPE (**5**) actually formed was detected as DOPEG (**2**) (Figure 3C). Although the yield of 2-oxo-DOPE (**5**) is very low, it is a major product in the early phase of the degradation of DOPEG (**2**)-quinone. Its low yield can be ascribed to the slow conversion from DOPEG (**2**)-quinone and the high reactivity of its quinone form, similar to the unstable DHBAld (**7**)-quinone (see below). It should also be noted that *ortho*-quinones of DOPE (**1**) and DOPEG (**2**) did not undergo a cyclization reaction to form a five-membered ring. This is in contrast to the formation of a six-membered ring from the *ortho*-quinone of rhododendrol through the hydroxy group [13].

When analyzed after ascorbic acid reduction, the oxidation of DOPAC (**3**) yielded 3,4-dihydroxybenzylalcohol (DHBAlc; **6**) as an initial and major product, which was gradually converted to DHBAld (**7**), although in low yield (**7**; Figure 3F). The identification of DHBAlc (**6**) and DHBAld (**7**) was confirmed by co-injection with authentic samples. The oxidation of DOMA (**4**) yielded DHBAld (**7**) as an initial and major product, which rapidly disappeared to give ill-defined polymeric product (Figure 2D and Figure 3H). The oxidation of DHBAld (**7**) itself afforded similar results on spectrophotometric and HPLC examinations (data not shown). The 3,4-dihydroxybenzoic acid, a possible product, was not detected. When analyzed after NaBH_4_ reduction, the DHBAld (**7**) actually formed was detected as DHBAlc (**6**) (Figure 3E,G).

In summary, 2-oxo-DOPE (**5**) was identified as the product of DOPEG (**2**)-quinone by ^1^H-NMR, ESI(+)/MS, and by co-injection on HPLC with the authentic sample provided by Manickam Sugumaran (Figures S5 and S6). The catechol products from DOPE (**1**)-quinone, DOPAC (**3**)-quinone, and DOMA (**4**)-quinone were identified with the authentic samples.

HPLC following oxidation was also performed at pH 5.3 to examine the effect of pH on the fate of *ortho*-quinones as DOPEG (**2**), DHBAlc (**6**) and DHBAld (**7**) by comparison. As shown in Figure S2, the *ortho*-quinones (analyzed as catechols after reduction) were much more stable at pH 5.3 than at pH 6.8, except for DHBAld (**7**) which degraded at similar rates at both pHs.

The half-lives of the *ortho*-quinones produced in this study are summarized in Table 1. The reduction with NaBH_4_ or ascorbic acid gave similar results with differences within 10% of the averages. The stability of *ortho*-quinones was in the order of DOPEG (**2**)-quinone > DOPE (**1**)-quinone > DHBAlc (**6**)-quinone > DHBAld (**7**)-quinone. These quinones were more reactive at pH 6.8 by a factor of 5–8 as compared to pH 5.3. An exception is that DHBAld (**7**)-quinone was as reactive at pH 5.3 as at pH 6.8.

## 3. Discussion

**Figure 4:**
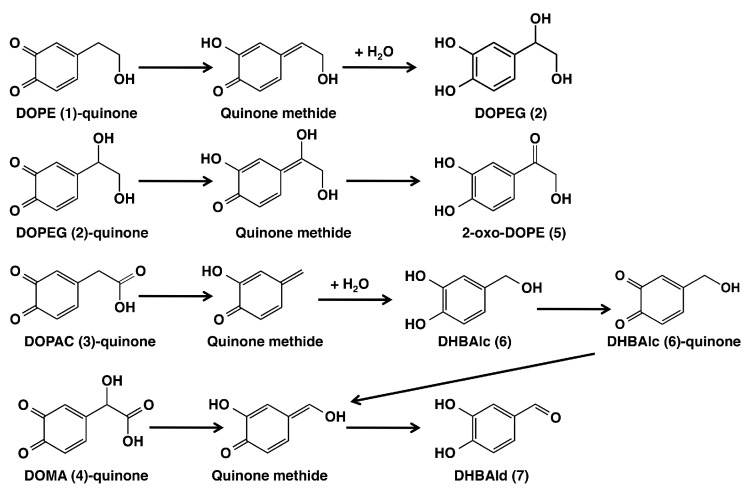
The metabolic fate of *ortho*-quinone products from catecholamine metabolites DOPE (**1**), DOPEG (**2**), DOPAC (**3**) and DOMA (**4**). The oxidation of DOPE (**1**) was stopped by the addition of NaBH_4_ or ascorbic acid (AA) to afford DOPEG (**2**). The oxidation of DOPEG (**2**) was stopped by the addition of AA to afford 2-oxo-DOPE (**5**). The oxidation of DOPAC (**3**) was stopped by the addition of AA to afford DHBAlc (**6**) and DHBAld (**7**). The oxidation of DOMA (**4**) was stopped by the addition of AA to afford DHBAld (**7**).

The catecholamines DA and NE are converted to alcoholic and acidic metabolites DOPE (**1**), DOPEG (**2**), DOPAC (**3**) and DOMA (**4**) in neurons (Figure 1). Our recent studies provided evidence for the incorporation of those catecholic metabolites into neuromelanins in the *substantia nigra* and the *locus coeruleus* in a pheomelanic structure [9,10]. This result implies the involvement of *ortho*-quinone intermediates that are trapped by cysteine to form cysteinyl derivatives [9,10,13]. In the present study, we used mushroom tyrosinase as an oxidant because it specifically and mildly oxidizes only catecholic substrates (and phenolic substrates) [15,16,20]. Our results show that the quinone methide intermediates are produced from the *ortho*-quinones of **1**–**4** through deprotonation in DOPE (**1**) and DOPEG (**2**) or through decarboxylation in DOPAC (**3**) and DOMA (**4**). Those quinone methides undergo the addition of a water molecule to form alcohols or tautomerization to form carbonyl compounds. These reaction pathways are summarized in Figure 4.

The metabolic fate of DOPE (**1**)-quinone was first reported by Sugumaran *et al.* [21] using a preparation of cuticular enzyme(s) from *Sarcophaga bullota*. With that insect enzyme(s) they were able to show a rapid conversion of DOPE (**1**)-quinone to DOPEG (**2**) through the quinone methide intermediate. DOPEG (**2**) was then gradually converted to 2-oxo-DOPE (**5**). However, that series of reactions did not proceed beyond the DOPE (**1**)-quinone stage when DOPE (**1**) was oxidized by mushroom tyrosinase [21]. The authors concluded that with the cuticular enzyme(s), quinone methide tautomerase acts on the *ortho*-quinone intermediate(s) to form quinone methide(s). The discrepancy between our results and theirs could be attributed to the fact that they followed the oxidation of DOPE (**1**) at a low concentration of tyrosinase while we followed the fate of DOPE (**1**)-quinone that was produced rapidly (<2 min) by a high concentration of tyrosinase. Later studies on the fate of DOPE (**1**)-quinone by d’Ischia’s group reported the production of a number of products, including dimers, but not DOPEG (**2**) [22,23]. The discrepancy may arise from differences in the oxidant (tyrosinase *vs.* peroxidase plus hydrogen peroxide), the concentration (0.1 *vs*. 1 mM) and/or the pH (6.8 *vs*. 7.4). The concentration is exceptionally important because the quinone methide formation is a first-order reaction that is independent of the concentration, whereas the dimer production is a concentration-dependent, second-order reaction.

The metabolic fate of DOPEG (**2**)-quinone has not been extensively studied except for the above-mentioned report by Sugumaran *et al.* [21]. In the present study, we identified the ketone metabolite of DOPEG (**2**), 2-oxo-DOPE (**5**), in the oxidation mixture when the oxidation was stopped by ascorbic acid (but not by NaBH_4_). The yield of this ketone was very low because of its high reactivity in the reaction mixture. In this connection, it should be mentioned that a similar, high reactivity of NE *ortho*-quinone yielded several side-chain breakdown products and a trimeric product, in addition to the cyclization products that eventually yield a melanic pigment [24,25].

It has been reported that DOMA (**4**)-quinone, generated either enzymatically [26,27] or electrochemically [28], is converted to DHBAld (**7**) through a decarboxylative rearrangement giving a quinone methide intermediate (Figure 4). The reaction proceeds extremely quickly with a half-life of 0.4 s [28]. DOPAC (**3**)-quinone also rapidly rearranges to give DHBAlc (**6**) through a quinone methide intermediate, and the product DHBAlc (**6**) is then gradually converted to DHBAld (**7**) through the same quinone methide as from DOMA (**4**)-quinone (Figure 4) [29,30]. The present study has confirmed the results of those previous studies and has characterized the half-lives of DHBAlc (**6**)-quinone and DHBAld (**7**)-quinone, which indicates a high reactivity of the latter with a half-life of 3 min.

DOPAC (**3**)-quinone and DOMA (**4**)-quinone do not undergo a cyclization reaction giving a five-membered lactone ring in this study. This is in sharp contrast to the behavior of the *ortho*-quinone of dihydrocaffeic acid (3,4-dihydroxyphenylpropionic acid), which cyclizes to give a six-membered lactone ring with a half-life of 9.9 min [31,32]. Sugumaran *et al.* [33,34] previously studied the fate of DOPAC (**3**)-quinone enzymatically generated and identified 2,5,6-trihydroxybenzofuran, a cyclization product, in addition to DOMA (**4**) and DHBAld (**7**). In our study DHBAlc (**6**) was nearly quantitatively produced at 2 min (Figure 3E,F). The reason for this discrepancy between their results and ours is not clear at present. However, one major difference in the reaction conditions is that we used a very high ratio of tyrosinase to substrate to generate DOPAC (**3**)-quinone within 1–2 min while they used a much lower ratio to generate it (>40 min).

The comparison of half-lives between pH 6.8 and 5.3 indicates that the quinone methide production from DOPE (**1**)-quinone, DOPEG (**2**)-quinone and DHBAlc (**6**)-quinone proceeds through the base-catalyzed mechanism as previously shown for 4-propyl-*o*-quinone by Bolton *et al.* [35]. It appears that the formation of quinone methides is a rather common pathway of *ortho*-quinone metabolism, even in the absence of quinone methide tautomerase [27,36]. For example, tyrosinase oxidation of 4-ethylphenol gives 1-(3,4-dihydroxyphenyl)ethanol as a major product [37]. These *ortho*-quinones may exert strong toxicity to neurons through binding with thiol enzymes and the production of reactive oxygen species *in vivo* [17,18,19]. In this regard, it should be worthwhile to point out that DHBAld (**7**)-quinone appears highly reactive as indicated by its short half-life of 3.0 min, the shortest among the *ortho*-quinones examined in this study (Table 1).

In summary, the fate of *ortho*-quinones derived from the catecholamine metabolites DOPE (**1**), DOPEG (**2**), DOPAC (**3**) and DOMA (**4**) provided evidence for the facile production of quinone methides as transient intermediates, which yielded stable products upon aromatization.

## 4. Experimental Section

### 4.1. Materials

Tyrosinase (from mushrooms, 1715 U/mg), 6-hydroxydopamine.HCl, DOPEG, DOPAC and DOMA were purchased from Sigma-Aldrich (St. Louis, MO, USA). The 3,4-dihydroxybenzaldehyde (DHBAld), 3,4-dihydroxybenzoic acid, ascorbic acid, NaBH_4_, NaIO_4_, formic acid, and methanol were purchased from Wako Pure Chemical Industry (Osaka, Japan). DOPE was purchased from Tokyo Chemical Industry (Tokyo, Japan). A solution of DHBAlc was prepared by reducing DHBAld with NaBH_4_ as described in 4.3. The 2-oxo-2-(3,4-dihydroxyphenyl)ethanol (2-oxo-DOPE) was a kind gift from Manickam Sugumaran (University of Massachusetts Boston, Boston, MA, USA).

### 4.2. Instruments

UV-visible spectra were measured with a JASCO V-630 UV-VIS spectrophotometer connected with an HMC-711 water thermostatted cell holder (JASCO Co., Tokyo, Japan). The HPLC system used to follow the course of tyrosinase oxidation consisted of a JASCO PU-2080 Plus pump (JASCO Co., Tokyo, Japan), a Shiseido C18 column (Capcell Pak MG; 4.6 × 250 mm; 5 µm particle size, Shiseido, Tokyo, Japan) and a JASCO UV-visible detector at 280 nm (JASCO Co., Tokyo, Japan). The mobile phases were 0.4 M formic acid: methanol, 90:10, 85:15 or 80:20 (*v*/*v*) (see Table 1). Analyses were performed at 35 or 50 °C (see Table 1) and a flow rate of 0.7 mL/min. For preparative HPLC to isolate 2-oxo-DOPE, a Shiseido C18 column (Capcell Pak MG; 20 × 250 mm; 5 µm particle size) was used with a mobile phase of 0.4 M formic acid:methanol (80:20 (*v*/*v*)) at 35 °C at a flow rate of 7.0 mL/min. The ^1^H NMR (400 MHz) spectrum was obtained with a Bruker AVANCE 400 spectrophotometer (Billerica, MA, USA) which was calibrated by adding methanol (3.34 ppm). Mass spectra were obtained using an Esquire HCT Plus Ion Trap Mass Spectrometer (mode: ESI) (Bruker, Billerica, MA, USA).

### 4.3. Oxidation of Catecholamine Metabolites with Mushroom Tyrosinase and HPLC Following Oxidation

Stock solutions of metabolites were prepared in ethanol or water at a 10 mM concentration. Test solutions of metabolites were prepared by diluting them to 100 µM with 50 mM sodium phosphate buffer (pH 6.8 or 5.3) when needed. A solution (2 mL) of 100 µM metabolite was oxidized by adding 20 µL (100 U) mushroom tyrosinase at 37 °C. The same amount of tyrosinase was also added to the reference cuvette. The oxidation was followed periodically up to 60 min with the UV-visible spectrophotometer. For HPLC following oxidation, a solution (1 mL) of 100 µM metabolite was oxidized by adding 20 µL (100 U) mushroom tyrosinase at 37 °C. The amount of tyrosinase was doubled in the HPLC analyses compared to the spectrophotometric analyses, so that oxidation of *ortho*-quinones completes faster, because we observed that oxidation of DOMA took several minutes to complete in the spectrophotometric analysis (Figure 2D). Aliquots of the reaction mixtures were periodically (2, 5, 10, 15, 30 and 60 min) withdrawn and subjected to analysis after reduction. For reductive termination of the reaction, 100 µL aliquots were mixed with 20 µL 10% NaBH_4_ or 5 mM ascorbic acid in water followed by the addition of 100 µL 0.8 M perchloric acid.

The half-lives (*t*_1/2_) of the *ortho*-quinones produced were calculated using the equation ln(c_0_/c) = *kt* and *kt*_1/2_ = 0.693.

### 4.4. Preparation of 2-Oxo-DOPE (**5**)

A solution of DOPEG (**2**) (17.0 mg, 0.1 mmol) in 100 mL 50 mM sodium phosphate buffer (pH 6.8) was gently shaken at 37 °C, to which NaIO_4_ (21.4 mg, 0.1 mmol) in 1 mL water was slowly added in 2 min. After 30 min, the mixture was poured into a solution of ascorbic acid (352 mg, 2 mmol) in 10 mL water. The mixture was extracted three times with ethyl acetate (100 mL each) and the combined ethyl acetate extract was dried over anhydrous sodium sulfate. The extract was evaporated under reduced pressure to give an oily residue, which was subjected to preparative HPLC to afford, after lyophilization, *ca.* 0.2 mg (HPLC purity, 98%; NMR purity, 91%) of 2-oxo-DOPE (**5**). Tyrosinase oxidation gave a lower yield of **5**. ^1^H NMR (400 MHz, CD_3_OD) δ 4.78 (2H, s, CH_2_), 6.81 (1H, d, *J* = 8.8 Hz, H-2’), 7.36 (1H, dd, *J* = 8.8, 2.0 Hz, H-6’), 7.37 (1H, d, *J* = 2.0 Hz, H-5’) (Figure S5). ESI(+)/MS: *m/z* 191.03 ([M + Na])^+^, 33%) 169.05 ([M + H]^+^, 100%) (Figure S6).

## 5. Conclusions

This study confirmed the fact that *ortho*-quinones from catechols **1**–**4** are converted to quinone methide tautomers as common intermediates, through proton rearrangement or decarboxylation. The unstable quinone methides afford stable alcoholic or carbonyl products (Figure 4). In *substantia nigra* and *locus coeruleus* neurons, the production of neuromelanin appears to detoxify otherwise toxic DA-quinone and NE-quinone [12,14]. Since it is likely that the formation of those *ortho*-quinones does not lead to incorporation into neuromelanin, the *ortho*-quinone of catecholamine metabolites, once formed, may exert cytotoxicity to neurons.

## Supplementary Materials

Supplementary materials can be found at http://www.mdpi.com/1422-0067/17/2/164/s1.

## References

[1] Zecca L., Bellei C., Costi P., Albertini A., Monzani E., Casella L., Gallorini M., Bergamaschi L., Moscatelli A., Turro N.J. (2008). New melanic pigments in the human brain that accumulate in aging and block environmental toxic metals. Proc. Natl. Acad. Sci. USA.

[2] Double K.L., Ben-Shachar D., Youdim M.B.H., Zecca L., Riederer P., Gerlach M. (2002). Influence of neuromelanin on oxidative pathways within the human substantia nigra. Neurotoxicol. Teratol..

[3] Mann D.M., Yates P.O. (1983). Possible role of neuromelanin in the pathogenesis of Parkinson’s disease. Mech. Ageing Dev..

[4] Marsden C.D. (1983). Neuromelanin and Parkinson’s disease. J. Neural. Transm. Suppl..

[5] Dias V., Junn E., Mouradian M.M. (2013). The role of oxidative stress in Parkinson’s disease. J. Parkinsons Dis..

[6] Zecca L., Stroppolo A., Gatti A., Tampellini D., Toscani M., Gallorini M., Giaveri G., Arosio P., Santambrogio P., Fariello R.G. (2004). The role of iron and copper molecules in the neuronal vulnerability of locus coeruleus and substantia nigra during aging. Proc. Natl. Acad. Sci. USA.

[7] Wakamatsu K., Fujikawa K., Zucca F.A., Zecca L., Ito S. (2002). The structure of neuromelanin as studied by chemical degradative methods. J. Neurochem..

[8] Wakamatsu K., Murase Y., Zucca F.A., Zecca L., Ito S. (2012). Biosynthetic pathway to neuromelanin and its aging process. Pigment. Cell Melanoma Res..

[9] Wakamatsu K., Tanaka H., Tabuchi K., Ojika M., Zucca F.A., Zecca L., Ito S. (2014). Reduction of the nitro group to amine by hydroiodic acid to synthesize *o*-aminophenol derivatives as putative degradative markers of neuromelanin. Molecules.

[10] Wakamatsu K., Tabuchi K., Ojika M., Zucca F.A., Zecca L., Ito S. (2015). Norephinephrine and its metabolites are involved in the synthesis of neuromelanin derived from the locus coeruleus. J. Neurochem..

[11] Eisenhofer G., Kopin I.J., Goldstein D.S. (2004). Catecholamine metabolism: A contemporary view with implications for physiology and medicine. Pharmacol. Rev..

[12] Napolitano A., Manini P., d’Ischia M. (2011). Oxidation chemistry of catecholamines and neuronal degeneration: An update. Curr. Med. Chem..

[13] Ito S., Wakamatsu K. (2008). Chemistry of mixed melanogenesis—Pivotal roles of dopaquinone. Photochem. Photobiol..

[14] Zecca L., Zucca F.A., Wilms H., Sulzer D. (2003). Neuromelanin of the substantia nigra: A neuronal black hole with protective and toxic characteristics. Trends Neurosci..

[15] Ito S., Yamashita T., Ojika M., Wakamatsu K. (2014). Tyrosinase-catalyzed oxidation of rhododendrol produces 2-methylchromane-6,7-dione, the putative ultimate toxic metabolite: Implications for melanocyte toxicity. Pigment. Cell Melanoma Res..

[16] Ito S., Wakamatsu K. (2015). A convenient screening method to differentiate phenolic skin whitening tyrosinase inhibitors from leukoderma-inducing phenols. J. Dermatol. Sci..

[17] Bolton J.L., Trush M.A., Penning T.M., Dryhurst G., Monks T.J. (2000). Role of quinones in toxicology. Chem. Res. Toxicol..

[18] Graham D.G., Tiffany S.M., Bell W.R., Gutknecht W.F. (1978). Autoxidation *versus* covalent binding of quinones as the mechanism of toxicity of dopamine, 6-hydroxydopamine, and related compounds toward C1300 neuroblastoma cells *in vitro*. Mol. Pharmacol..

[19] Ito S., Okura M., Nakanishi Y., Ojika M., Wakamatsu K., Yamashita T. (2015). Tyrosinase-catalyzed metabolism of rhododendrol (RD) in B16 melanoma cells: Production of RD-pheomelanin and covalent binding with thiol proteins. Pigment. Cell Melanoma Res..

[20] Ramsden C.A., Riley P.A. (2014). Tyrosinase: The four oxidation states of the active site and their relevance to enzymatic activation, oxidation and inactivation. Bioorg. Med. Chem..

[21] Sugumaran M., Semensi V., Saul S.J. (1989). On the oxidation of 3,4-dihydroxyphenyl alcohol and 3,4-dihydroxyphenyl glycol by cuticular enzyme(s) from *Sarcophaga bullata*. Arch. Insect Biochem. Physiol..

[22] Vogna D., Pezzella A., Panzella L., Napolitano A., d’Ischia M. (2003). Oxidative chemistry of hydroxytyrosol: isolation and characterization of novel methanooxocinobenzodioxinone derivatives. Tetrahedron Lett..

[23] De Lucia M., Panzella L., Pezzella A., Napolitano A., d’Ischia M. (2006). Oxidative chemistry of the natural antioxidant hydroxytyrosol: Hydrogen peroxide-dependent hydroxylation and hydroxyquinone/*o*-quinone coupling pathways. Tetrahedron.

[24] Manini P., Pezzella A., Panzella L., Napolitano A., d’Ischia M. (2005). New insight into the oxidative chemistry of noradrenaline: Competitive *o*-quinone cyclisation and chain fission routes leading to an unusual 4-[bis-(1*H*-5,6-dihydroxyindol-2-yl)methyl]-1,2-dihydroxybenzene derivative. Tetrahedron.

[25] Manini P., Panzella L., Napolitano A., d’Ischia M. (2007). Oxidation chemistry of norepinephrine: Partitioning of the *o*-quinone between competing cyclization and chain breakdown pathways and their roles in melanin formation. Chem. Res. Toxicol..

[26] Sugumaran M. (1986). Tyrosinase catalyzes an unusual oxidative decarboxylation of 3,4-dihydroxymandelate. Biochemistry.

[27] Sugumaran M., Dali H., Semensi V. (1992). Mechanistic studies on tyrosinase-catalysed oxidative decarboxylation of 3,4-dihydroxymandelic acid. Biochem. J..

[28] Czapla T.H., Claeys M.R., Morgan T.D., Kramer K.J., Hopkins T.L., Hawley M.D. (1991). Oxidative decarboxylation of 3,4-dihydroxymandelic acid to 3,4-dihydroxybenzaldehyde: Electrochemical and HPLC analysis of the reaction mechanism. Biochim. Biophys. Acta.

[29] Mefford I.N., Kincl L., Dykstra K.H., Simpson J.T., Markey S.P., Dietz S., Wightman R.M. (1996). Facile oxidative decarboxylation of 3,4-dihydroxyphenylacetic acid catalyzed by copper and manganese ions. Biochim. Biophys. Acta.

[30] Sugumaran M., Semensi V., Dali H., Nellaiappan K. (1991). Oxidation of 3,4-dihydroxybenzyl alcohol: A sclerotizing precursor for cochroach ootheca. Arch. Insect Biochem. Physiol..

[31] Sugumara M., Dali H., Kundzicz H., Semensi V. (1989). Unusual, intramolecular cyclization and side chain desaturation of carboxyethyl-*o*-benzoquinone derivatives. Bioorg. Chem..

[32] Moridani M.Y., Scobie H., Jamshidzadeh A., Salehi P., O’Brien P.J. (2001). Caffeic acid, chlorogenic acid, dihydrocaffeic acid metabolism: Glutathione conjugate formation. Drug Metab. Dispos..

[33] Sugumara M., Semensi V., Dali H., Mitchell W. (1989). Novel transformations of enzymatically generated carboxymethyl-*o*-benzoquinone to 2,5,6-trihydroxybenzofuran and 3,4-dihydroxymandelic acid. Bioorg. Chem..

[34] Sugumaran M., Duggaraju P., Jayachandran E., Kirk K.L. (1999). Formation of a new quinone methide intermediate during the oxidative transformation of 3,4-dihydroxyphenylacetic acids: Implication for eumelanin biosynthesis. Arch. Biochem. Biophys..

[35] Bolton J.L., Wu H.M., Hu L.Q. (1996). Mechanism of isomerization of 4-propyl-*o*-quinone to its tautomeric *p*-quinone methide. Chem. Res. Toxicol..

[36] Thompson D.C., Thompson J.A., Sugumaran M., Moldéus P. (1993). Biological and toxicological consequences of quinone methide formation. Chem. Biol. Interact..

[37] Brooks S.J., Nikodinovic J., Martin L., Doyle E.M., O’Sullivan T., Guiry P.J., Coulombel L., Li Z., O’Connor K.E. (2013). Production of a chiral alcohol, 1-(3,4-dihydroxyphenyl)ethanol, by mushroom tyrosinase. Biotechnol. Lett..

