# Supplementary material for: The Metabolic Fate of ortho-Quinones Derived from Catecholamine Metabolites
Source: Int J Mol Sci. 2016 Jan 27;17(2):164. doi: 10.3390/ijms17020164 (PMC4783898; doi:10.3390/ijms17020164)
Supplement: Supplementary file 1 [file ijms-17-00164-s001.pdf]

Supplementary Materials: The Metabolic Fate of *Ortho*-Quinones Derived from Catecholamine Metabolites

Shosuke Ito, Yuta Yamanaka, Makoto Ojika and Kazumasa Wakamatsu

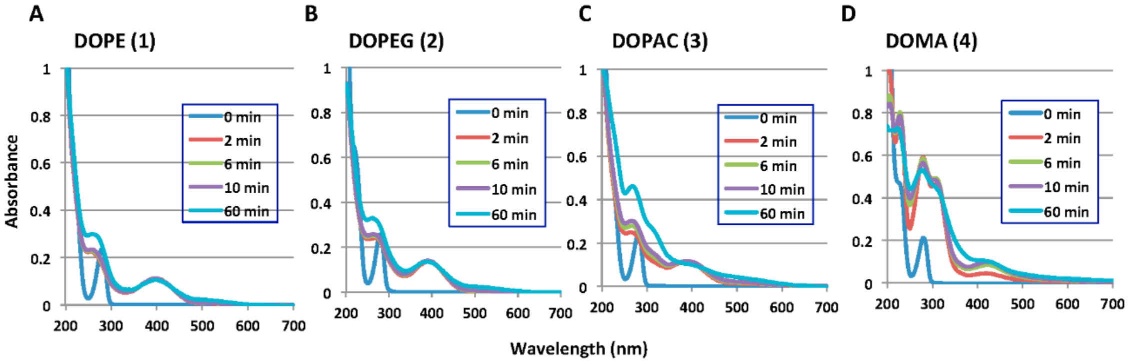

Figure S1. Time course of the degrada1on of *ortho*-quinone products from catecholamine metabolites at pH 5.3. (**A**) DOPE (**1**); (**B**) DOPEG (**2**); (**C**) DOPAC (**3**); (**D**) DOMA. Each experiment was performed twice with similar results.

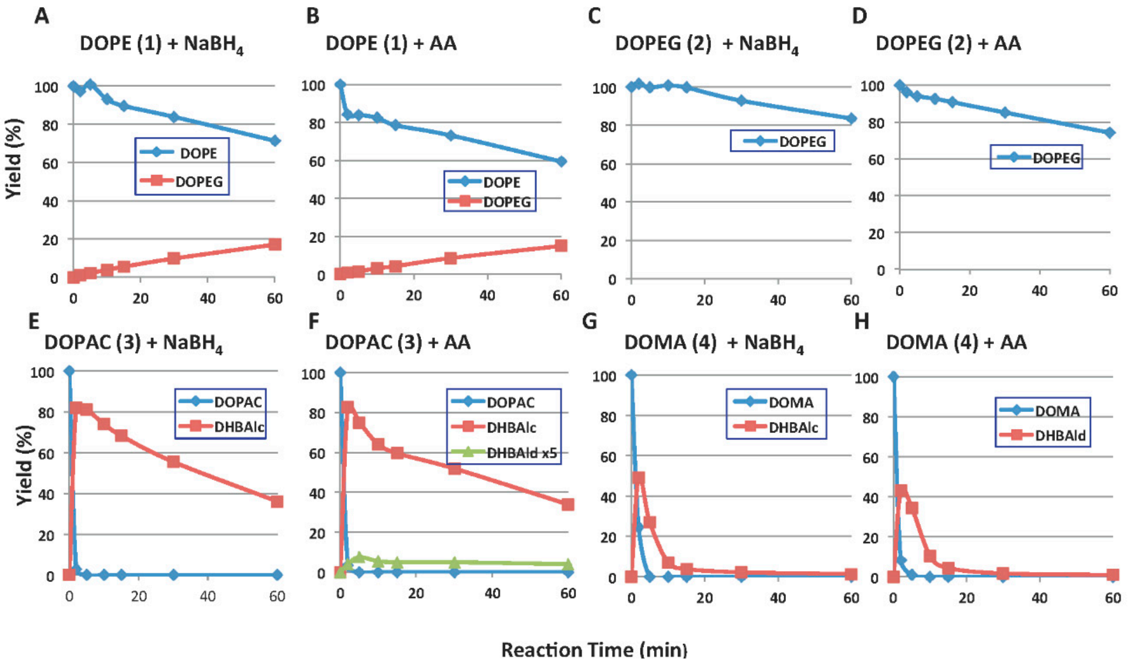

Figure S2. HPLC following the degrada1on of *ortho*-quinone products from catecholamine metabolites at pH 5.3. (**A**,**B**) DOPE (**1**); (**C**,**D**) DOPEG (**2**); (**E**,**F**) DOPAC (**3**); (**G**,**H**) DOMA. For **A**, **C**, **E** and **G**, the oxida1on was stopped by the addi1on of $NaBH_4$; For **B**, **D**, **F** and **H**, the oxida1on was stopped by the addi1on of ascorbic acid. For the sake of clarity, the yields of DHBAld (**7**) were mul1plied by a factor of 5 in (**F**).

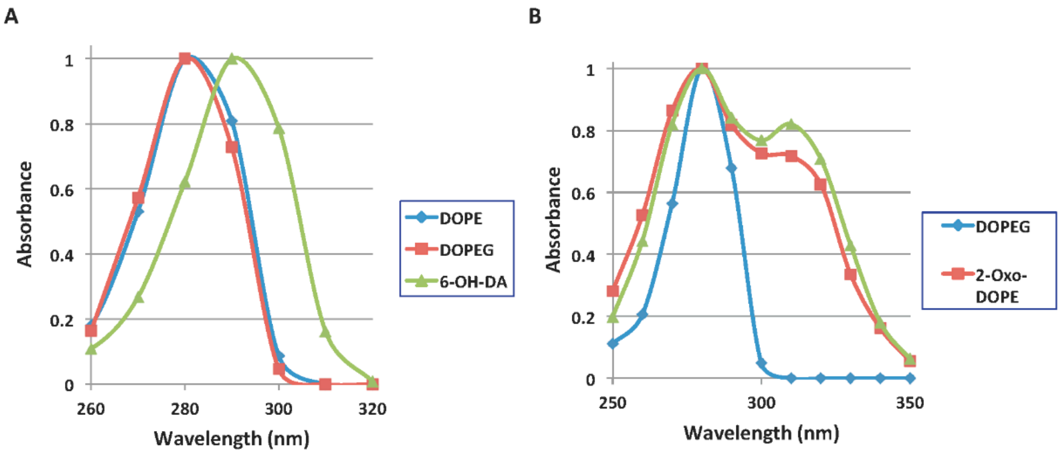

Figure S3. UV absorp1on spectra of DOPEG (**2**) and 2-oxo-DOPE (**5**) obtained by oxida1on of DOPE (**1**) and DOPEG (**2**), respec1vely. (**A**) Spectrum of DOPEG (**2**) obtained from DOPE (**1**) in comparison with those of DOPE and 6-hydroxydopamine; (**B**) Spectrum of 2-oxo-DOPE (**5**) obtained from DOPEG (**2**) in comparison with those of DOPEG and DHBAld (**7**). The spectrum of 6-hydroxydopamine was taken with the UV-VIS spectrophotometer, while the others were obtained from HPLC chromatograms analyzed for the wavelength indicated. Maximal absorbance values were normalized to 1.

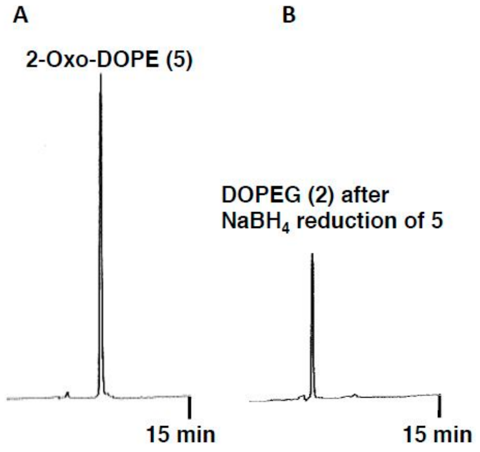

Figure S4. HPLC chromatograms of (**A**) 2-oxo-DOPE (**5**) and (**B**) DOPEG (**2**) obtained after the $NaBH_4$ reduc1on of 2-oxo-DOPE. The same molar quan11es were injected for **A** and **B**.

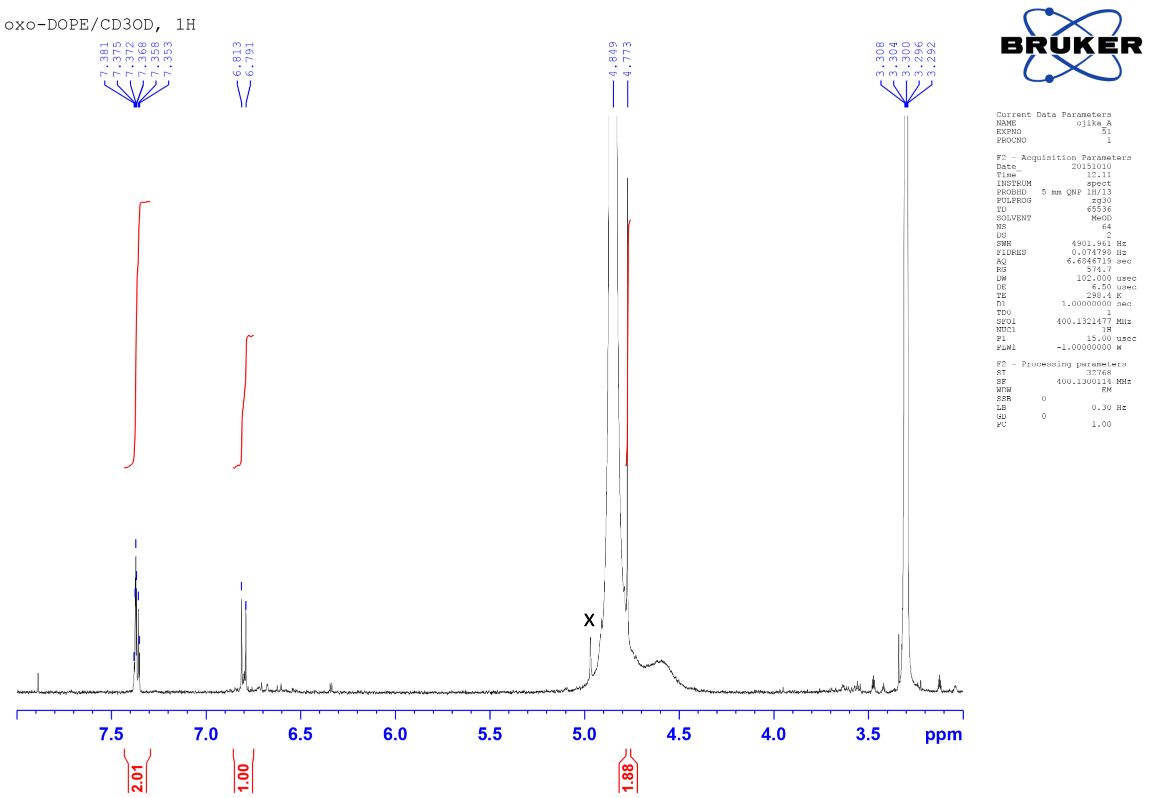

Figure S5. ^{1}H-NMR spectrum of 2-oxo-DOPE (**5**). X is a peak derived from impurity.

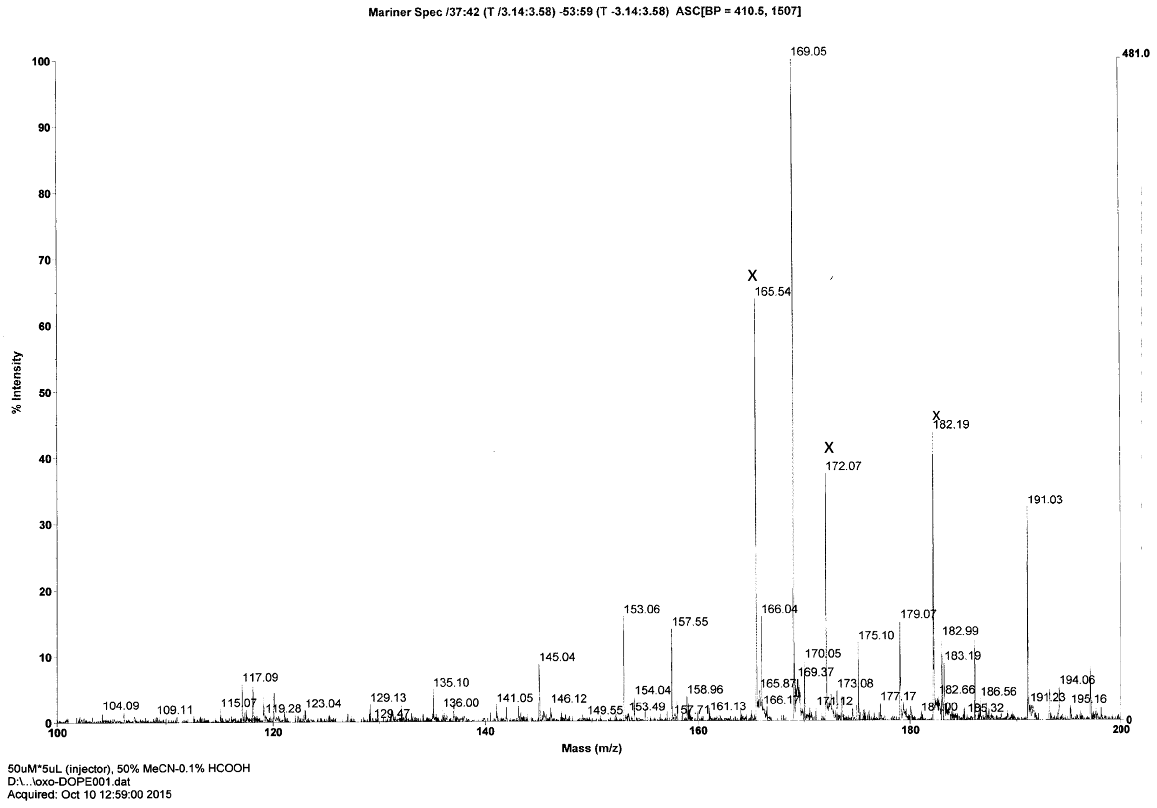

Figure S6. ESI(+)/MS spectrum of 2-oxo-DOPE (**5**). X is a peak derived from impurity.
